# Altered Plasma Apolipoprotein Modifications in Patients with Pancreatic Cancer: Protein Characterization and Multi-Institutional Validation

**DOI:** 10.1371/journal.pone.0046908

**Published:** 2012-10-08

**Authors:** Kazufumi Honda, Takuji Okusaka, Klaus Felix, Shoji Nakamori, Naohiro Sata, Hideo Nagai, Tatsuya Ioka, Akihiko Tsuchida, Takeshi Shimahara, Masashi Shimahara, Yohichi Yasunami, Hideya Kuwabara, Tomohiro Sakuma, Yoshihiko Otsuka, Norihito Ota, Miki Shitashige, Tomoo Kosuge, Markus W. Büchler, Tesshi Yamada

**Affiliations:** 1 Division of Chemotherapy and Clinical Research, National Cancer Center Research Institute, Tokyo, Japan; 2 Hepatobiliary and Pancreatic Oncology Division, National Cancer Center Hospital, Tokyo, Japan; 3 Department of Surgery, University of Heidelberg, Heidelberg, Germany; 4 Department of Surgery, Osaka National Hospital, National Hospital Organization, Osaka, Japan; 5 Department of Surgery, Jichi Medical University, Shimotsuke, Japan; 6 Department of Hepatobiliary and Pancreatic Oncology, Osaka Medical Center for Cancer and Cardiovascular Diseases, Osaka, Japan; 7 Third Department of Surgery, Tokyo Medical University, Tokyo, Japan; 8 Department of Oral Surgery, Osaka Medical College, Osaka, Japan; 9 Department of Regenerative Medicine and Transplantation, Fukuoka University Faculty of Medicine, Fukuoka, Japan; 10 BioBusiness Group, Mitsui Knowledge Industry, Tokyo, Japan; 11 Pancreatic Cancer Diagnosis Project, Molecuence Corporation, Yokohama, Japan; 12 Hepatobiliary and Pancreatic Surgery Division, National Cancer Center Hospital, Tokyo, Japan; Technische Universität München, Germany

## Abstract

**Background:**

Among the more common human malignancies, invasive ductal carcinoma of the pancreas has the worst prognosis. The poor outcome seems to be attributable to difficulty in early detection.

**Methods:**

We compared the plasma protein profiles of 112 pancreatic cancer patients with those of 103 sex- and age-matched healthy controls (Cohort 1) using a newly developed matrix-assisted laser desorption/ionization (oMALDI) QqTOF (quadrupole time-of-flight) mass spectrometry (MS) system.

**Results:**

We found that hemi-truncated apolipoprotein AII dimer (ApoAII-2; 17252 *m/z*), unglycosylated apolipoprotein CIII (ApoCIII-0; 8766 *m/z*), and their summed value were significantly decreased in the pancreatic cancer patients [*P* = 1.36×10^−21^, *P* = 4.35×10^−14^, and *P* = 1.83×10^−24^ (Mann-Whitney *U*-test); area-under-curve values of 0.877, 0.798, and 0.903, respectively]. The significance was further validated in a total of 1099 plasma/serum samples, consisting of 2 retrospective cohorts [Cohort 2 (*n* = 103) and Cohort 3 (*n* = 163)] and a prospective cohort [Cohort 4 (*n* = 833)] collected from 8 medical institutions in Japan and Germany.

**Conclusions:**

We have constructed a robust quantitative MS profiling system and used it to validate alterations of modified apolipoproteins in multiple cohorts of patients with pancreatic cancer.

## Introduction

With a 5-year survival rate of less than 10%, invasive ductal carcinoma of the pancreas has the worst prognosis among the more common human malignancies [1], [2], [3]. The poor outcome of pancreatic cancer patients seems to be attributable to difficulty in early detection. Most patients with early-stage pancreatic cancer have no symptoms, and do not visit clinics until the disease has progressed to an advanced stage. Because surgical resection is currently the only curative treatment for pancreatic cancer, early detection before the development of metastasis is essential to improve its outcome. However, no screening method has been established for pancreatic cancer [2]. Enhanced computed tomography (CT) and positron emission tomography (PET) are useful for the diagnosis of pancreatic diseases, but these modalities are potentially hazardous and would probably be too labor-intensive and cost-ineffective for mass screening, because of the relatively low incidence of pancreatic cancer.

The circulating blood proteome holds great promise as a reservoir of information that could be used for the diagnosis of various physiological and pathological conditions [4]. We previously found a plasma biomarker set that was able to distinguish pancreatic cancer patients including those with stage I and II disease from healthy individuals by mass spectrometry (MS)-based proteomics [5]. However, it was not possible to identify the marker proteins, and the numbers/sources of cases and types of disease were limited [6].

In the present study, we determined the amino acid sequences and modifications of marker proteins and validated their significance in larger cohorts. Here we report that the two modified forms of plasma/serum apolipoproteins are reduced in patients with pancreatic diseases.

## Materials and Methods

### Patient Samples

Four cohorts (namely Cohorts 1–4) consisting of a total of 1314 plasma or serum samples were collected at the following medical institutions in two countries (Japan and Germany) (Tables S1 and S2):

#### Cohort 1

215 plasma samples [sex- and age-matched patients with histologically or cytologically proven pancreatic ductal adenocarcinoma (*n* = 103) and healthy controls (*n* = 112)] collected at the National Cancer Center Hospital (NCCH) (Tokyo, Japan) and Tokyo Medical University Hospital (TMUH) (Tokyo, Japan) between August 2002 and February 2005, as reported previously [5].

#### Cohort 2

103 plasma samples [sex- and age-matched pancreatic cancer patients (*n* = 62) and healthy controls (*n* = 41)] newly collected at the NCCH between August 2003 and May 2005.

#### Cohort 3

163 serum samples [pancreatic cancer patients (*n* = 52), patients with chronic pancreatitis (*n* = 58) and healthy controls (*n* = 53)] collected at the Department of General Surgery, University of Heidelberg (Heidelberg, Germany) between 2003 and 2006 [7], [8].

#### Cohort 4

833 plasma (and serum) samples collected prospectively from 7 medical institutions in Japan [NCCH, Osaka National Hospital (Osaka, Japan), Jichi Medical School Hospital (Shimotsuke, Japan), Osaka Medical Center for Cancer and Cardiovascular Diseases (Osaka, Japan), TMUH, Osaka Medical College Hospital (Osaka, Japan), and Fukuoka University Hospital (Fukuoka, Japan)] between August 2006 and October 2008 for this study. A document detailing standard operating procedures was distributed to each institute, and all the plasma samples were collected prospectively under identical conditions. This was an essentially hospital-based cohort consisting of healthy volunteers and newcomers to mainly gastrointestinal services at the participating hospitals. The final diagnoses of the patients were collected separately from their blood samples. Written informed consent was obtained from every subject. The study protocol was reviewed and approved by the ethics committee of each participating institution.

The collection and storage conditions (such as collection tubes, anticoagulants, temperature, and number of thawing and freezing cycles) were kept strictly identical among plasma samples within the same cohort. Apparently hemolyzed samples were excluded. All the samples were blinded and randomized prior to MS analysis (detailed procedures are available in Methods S1). Only plasma samples were used for the MS analysis of Cohort 4. To ensure reproducibility of measurements, the samples in Cohort 4 were analyzed independently by two investigating teams at different laboratories [NCCRI (Tokyo, Japan) and Molecuence Corporation (Yokohama, Japan)].

### Immunoprecipitation and Immunoblotting

Anti-pan ApoAII rabbit polyclonal antibody was purchased from Abcam (Cambridge, UK). Immunoprecipitation was performed using immunoaffinity chromatography (IAC) Protein G beads (Bruker Daltonics). Two rabbit polyclonal antibodies were raised against ApoAII monomer peptides with -ATQ and -AT C-termini. cDNA fragments of ApoAII (encoding amino acids 1–100 and 1–99; NP_001634) were amplified by PCR and subcloned into pGEX-6P-2 plasmids (GE Healthcare). N-terminally glutathione S-transferase (GST)-tagged fusion proteins were expressed in *Escherichia coli* and affinity purified on glutathione-Sepharose 4B (GE Healthcare) as described previously [9]. The reactivity of antibodies with the GST-tagged proteins was assessed as described previously [10].

Protein samples were separated by SDS-PAGE and electroblotted onto polyvinylidene difluoride membranes (Millipore, Billerica, MA). Blots were visualized with an enhanced chemiluminescence kit (GE Healthcare) and quantified as described previously [11].

### CA19-9 Measurement

Serum samples in Cohort 4 were analyzed using commercially available chemiluminescence enzyme immunoassay kits for CA19-9 (LUMIPULSE Presto CA19-9, Fujirebio Inc., Tokyo, Japan) [5].

### Statistics

Statistically significant differences were detected using Mann-Whitney *U*-, Student *t*-, and χ^2^ tests. Receiver operator characteristic (ROC) curves were generated and AUC values were calculated using StatFlexs software (version 5.0; Artech, Osaka, Japan) and the modules of R-project (http://www.r-project.org/) [5], [12], [13].

## Results

### Development of MS Measurement with High Reproducibility

**Figure 1:**
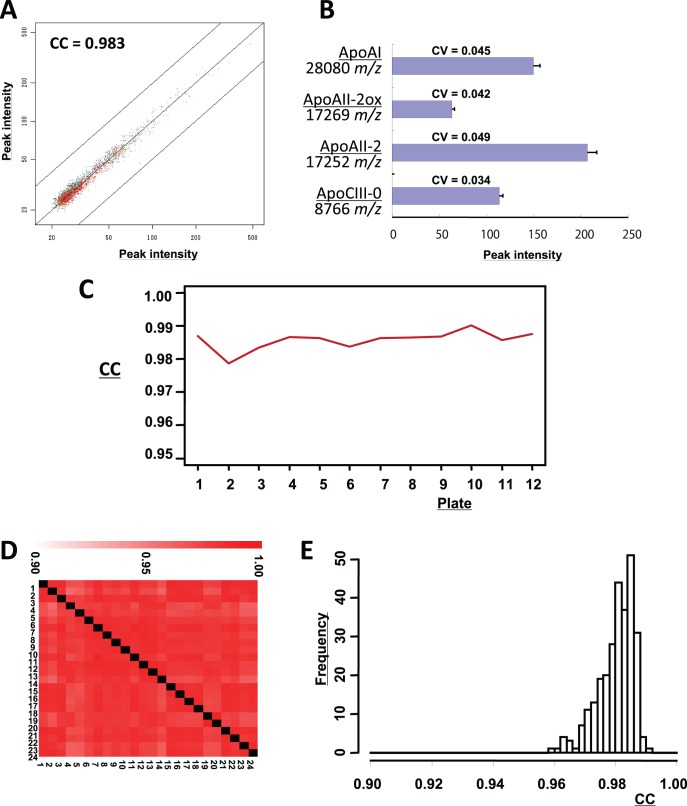
Reproducibility of automated chromatography and oMALDI-QqTOF-MS. (**A**) Two-dimensional plot analysis showing the correlation of 2173 corresponding peaks between two of the triplicate measurements (indicated in red, blue, and green) of a standard plasma mixture. Lines indicate 2-fold differences. (**B**) A standard plasma mixture was analyzed 24 times, and the CV values [ =  SD (bars)/mean (columns)] of representative protein peaks (ApoAI, ApoAII-2ox, ApoAII-2, and ApoCIII-0) were calculated. (**C**) Transition of CC values between the duplicate measurements of a standard plasma mixture in the 12 plates (24 measurements) of Cohort 1. (**D and E**) Correlation matrix (**D**) and distribution (**E**) of mutual similarity (CC values) for the 24 duplicate measurements (1–24).18.

We developed a plasma protein profiling system by employing automated hydrophobic chromatography and oMALDI QqTOF-MS. In a quality control experiment, 2173 independent low-molecular-weight (within the range 1000–30,000 *m/z*) protein peaks were detected from a 5-µl sample of mixed plasma from healthy volunteers. The average correlation coefficient (CC) value for all 2173 peaks among triplicate measurements was 0.983 (Fig. 1A). The reproducibility of measurement was further confirmed by calculating the coefficient of variation (CV) values for peaks of interest (see below) (Fig. 1B).

We prepared a large stock of plasma mixture as a quality control standard, and two aliquots of the stock were processed simultaneously with every 22 test samples. The CC value of the standard was monitored to ensure consistency of measurement for every plate (Fig. 1C–E).

### Protein Identification by Tandem Mass Spectrometry (MS/MS)

**Figure 2:**
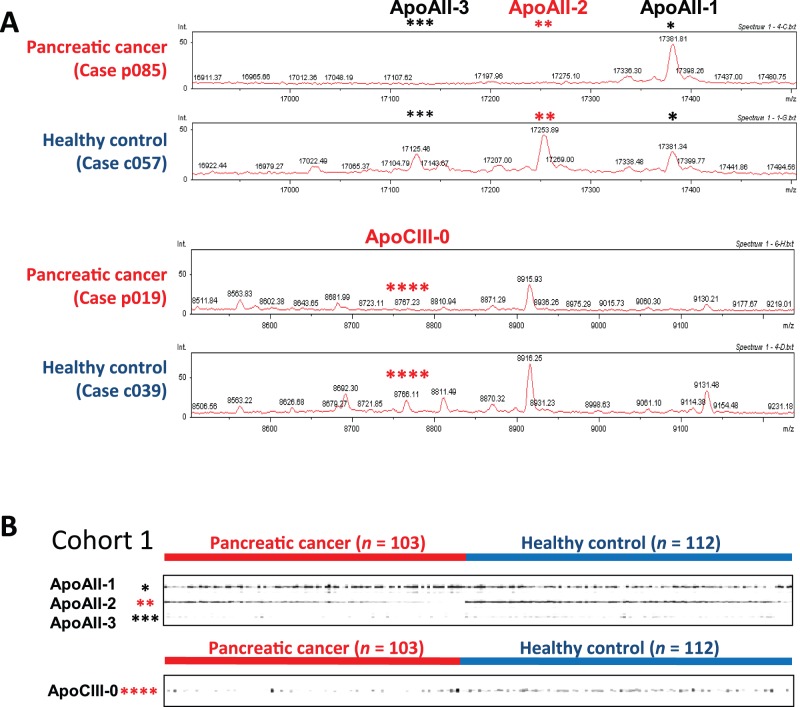
oMALDI-QqTOF-MS profiling of plasma proteins. (**A**) Spectra for representative pancreatic cancer patients and controls within the ranges 16,900 to 17,500 *m/z* (top) and 8500 to 9250 *m/z* (bottom). (**B**) Gel-like images of ApoAII and ApoCIII-0 in Cohort 1 (215 cases). (**A** and **B**) Black single asterisks (*), red double asterisks (**), black triple asterisks (***), and red quadruple asterisks (****) indicate MS peaks of ApoAII-1, ApoAII-2, ApoAII-3, and ApoCIII-0, respectively.

Quantitative plasma MS data were obtained from 103 patients with pancreatic cancer and 112 sex- and age-matched healthy controls (Cohort 1). We found a 17252-*m/z* MS peak (Fig. 2, indicated by double asterisks) that stood out as having the highest statistical significance (*P* = 1.36×10^−21^, Mann-Whitney *U*-test) (Fig. S1). The molecular weight was almost identical to that of a marker protein we had identified previously using a different MS system [5]. MS/MS analysis and database/literature searches revealed that the 17252-m/z protein was a heterodimer of ApoAII, one peptide chain of which lacked a glutamine (Q) residue at the C-terminus (-ATQ/−AT) (namely, ApoAII-2) (Figs. S2 and S3) [7], [14]. The oxidized form of ApoAII-2 (17269 *m/z*, ApoAII-2ox) [14] showed the second highest statistical significance (*P* = 5.14×10^−20^) (Table S3).

**Figure 3:**
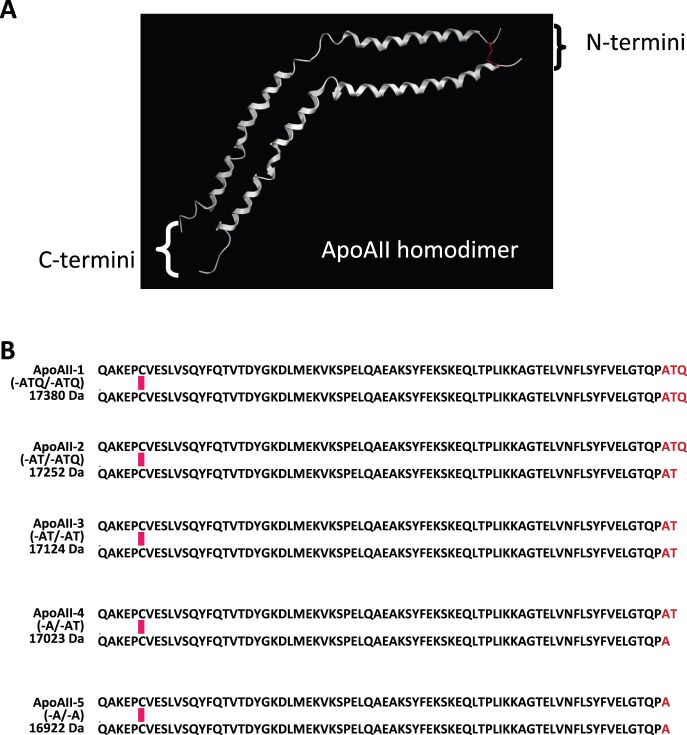
Structure of ApoAII dimers. (**A**) Predicted three-dimensional structure of dimerized ApoAII protein. The model was built using the MOE software package (Ryoka Systems Inc., Tokyo, Japan). Red color indicates a disulfide bond. (**B**) Amino acid sequences and calculated theoretical molecular masses of ApoAII homo/heterodimers (ApoAII-1 to -5). Two peptides were dimerized via an N-terminal disulfide bond.

In order to confirm the identity of the protein, we carried out immunoprecipitation with an antibody specific to ApoAII, and the immunoprecipitated proteins were analyzed by oMALDI-QqTOF-MS (Fig. S4). ApoAII-2 (-ATQ/−AT) and its oxidized form (ApoAII-2ox) were detected along with other post-translationally modified forms of ApoAII [AII-1 (17380 *m/z*) and AII-3 (17124 *m/z*)]. ApoAII-1 is a homodimer of untruncated ApoAII peptides with C-terminal ends of -ATQ/−ATQ. ApoAII-3 is a homodimer of AII peptide chains, both the C-terminal ends of which lack glutamine (Q) residues (-AT/−AT) (Fig. 3) [14].

Another protein with a molecular size of 8766 *m/z* also showed a highly significant difference (*P* = 4.35×10^−14^) between pancreatic cancer patients and healthy controls (Fig. 2, indicated by quadruple asterisks). The molecular size of the protein was also almost identical to that of one of 4 marker proteins that we had reported in our previous study [5]. MS/MS analysis (Fig. S5) identified the 8766-*m/z* protein as ApoCIII, which is a 79-amino-acid glycoprotein known to have three differently glycosylated isoforms {unglycosylated (CIII-0), monosialyated (CIII-2), and disialylated (CIII-2) [15], [16]}, and the 8766-*m/z* protein corresponds to ApoCIII-0. The identity was confirmed by MS analysis of proteins immunoprecipitated with anti-ApoCIII antibody (data not shown).

### Combination of ApoAII-2 and ApoCIII-0 and its Validation

**Figure 4:**
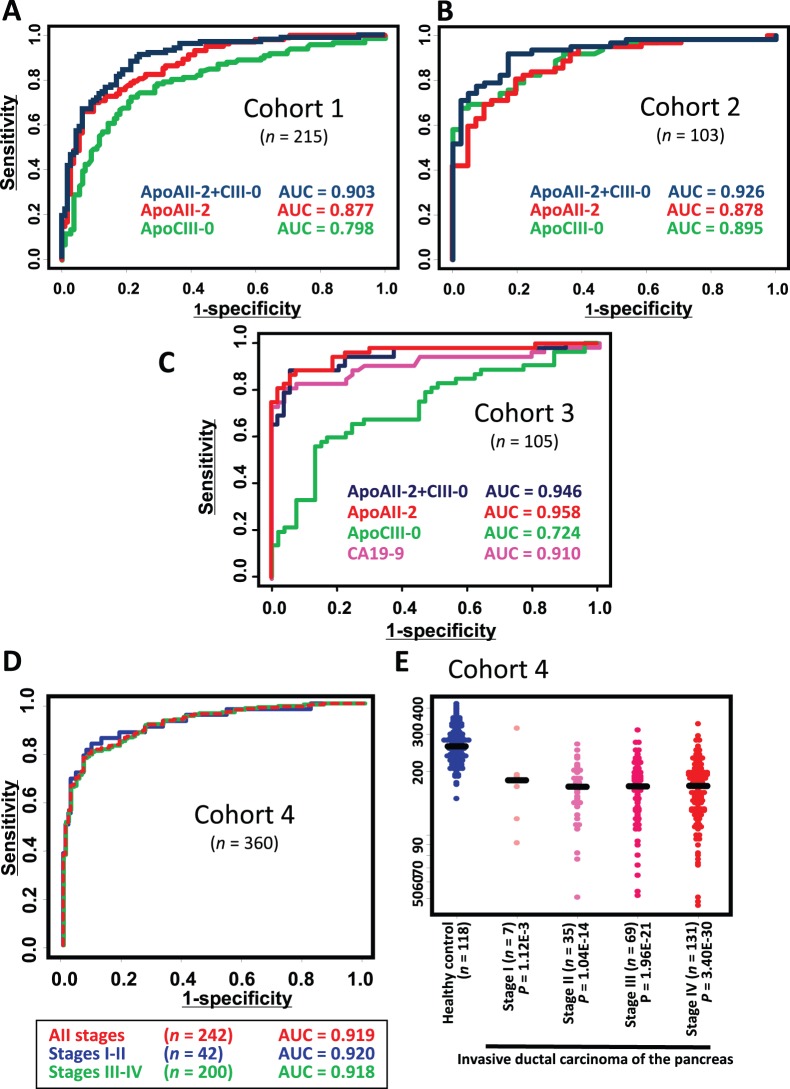
Reduction of ApoAII-2 and ApoCIII-0 in pancreatic cancer. (**A and B**) ROC analysis of ApoAII-2, ApoCIII-0, and their combination (ApoAII-2+CIII-0) in Cohorts 1 (A) and 2 (B). (**C**) ROC analysis showing the capacity of ApoAII-2, ApoCIII-0, ApoAII-2+CIII-0, and CA19-9 for discrimination of pancreatic cancer patients from healthy controls in the German cohort (Cohort 3). (**D**) ROC analysis of ApoAII-2+CIII-0 in Cohort 4 according to clinical stage (Cohort 4). (**E**) Distribution of ApoAII-2+CIII-0 according to clinical stage. Lines indicate median values (Cohort 4).

ApoAII-2 and ApoCIII-0 were able to differentiate the 103 pancreatic cancer patients from the 112 healthy controls (Cohort 1) with an area-under-curve (AUC) value of 0.877 and 0.798, respectively (Fig. 4A and Table S3). Because both ApoAII-2 and ApoCIII-0 were decreased in pancreatic cancer patients and the distribution was mutually independent (Fig. S6), simple summing of the two biomarkers improved the AUC value to 0.903.

The high discrimination capability of ApoAII-2, ApoCIII-0, and their summed value (hereafter denoted as ApoAII-2+CIII-0) between pancreatic cancer patients and controls was validated in an independent cohort consisting of 41 sex- and age-matched healthy controls and 62 pancreatic cancer patients (Cohort 2) (Fig. 4B and Table S3).

To confirm the universality of this discrimination across different ethnicities, we next examined 163 serum samples collected in Germany (Cohort 3) (Fig. 4C and Table S4) [7], [8]. The reduction of ApoAII-2 and ApoCIII-0 in pancreatic cancer patients was evident even in this cohort. However, the decrease of ApoAII-2 and ApoCIII-0 was found not to be specific to pancreatic cancer. ApoAII-2+CIII-0 was decreased significantly in patients with chronic pancreatitis (*P* = 8.86×10^−11^).

The mean age of healthy controls in Cohort 3 (39.1 years) was significantly lower that that of patients with pancreatic cancer (63.1 years) or pancreatitis (50.3 years) (Table S1). Therefore, the possibility that the extremely high value of ApoAII-2 (0.958) in this cohort was attributable to this age difference cannot be excluded.

### Prospective Multi-institutional Validation

Plasma/serum protein profiling by direct MS was introduced as a revolutionary new tool for biomarker discovery [17], [18]. However, its validity has been frequently discussed [6], [19], mainly on the basis of possible biases in sample collection, storage, and freeze/thawing, as well as poor mass accuracy, thus creating difficulty with protein identification. To ensure the absence of any sampling bias, we then collected plasma samples prospectively from 7 medical institutions in Japan (Cohort 4) using the same predetermined protocol of blood collection, storage, and freeze/thawing. This multi-institutional collaborative study group was organized by the “Third-Term Comprehensive Control Research for Cancer" conducted by the MHLWJ and affiliated to the International Cancer Biomarker Consortium (ICBC) (http://www.fhcrc.org/science/international_biomarker/) and follows the principle of PRoBE design (prospective specimen collection before outcome ascertainment and retrospective blinded evaluation).

All the prospectively collected samples were blinded, randomized, and sent to a laboratory at the Molecuence Corporation. Even this independent/blinded validation showed that pancreatic cancer patients, including those with early-stage disease, were readily distinguishable from healthy controls using the combination of ApoAII-2 and ApoCIII-0 (ApoAII-2+CIII-0). The AUC values for ApoAII-2+CIII-0 were 0.920, 0.918, and 0.919 for pancreatic cancer patients at clinical stages I-II, III-IV, and all stages [TNM classification, UICC (International Union against Cancer)], respectively (Fig. 4D).

### Comparison and Combination with CA19-9

The combination of ApoAII-2 and ApoCIII-0 (ApoAII-2+CIII-0) was able to detect 66.1% (160/242) of pancreatic cancer patients relative to healthy individuals (Table S5) at a cut-off value [189 arbitrary units (AU)] that had been arbitrarily determined to obtain a specificity of 95% or higher [97.5% (115/118)]. With this cut-off, the sensitivity of ApoAII-2+CIII-0 became inferior to that of CA19-9 [79.8% (193/242)], but the specificity was superior to that of CA19-9 [94.9% (112/118)], and the AUC value of ApoAII-2+CIII-0 was almost equal to that of CA19-9. However, the reduction of ApoAII-2+CIII-0 was not influenced by the clinical stage of the disease, and was detectable even in patients with stage I pancreatic cancer (Fig. 4E). The AUC value of ApoAII-2+ApoCIII0 for stage I pancreatic cancer (0.868) was higher than that of CA19-9 (0.774) (Table S5), suggesting the potential advantage of ApoAII-2+ApoCIII0 over CA19-9 for detection of early-stage pancreatic cancer. However, the number of stage I pancreatic cancer patients examined in this study was still small (7 cases), and this issue remains to be confirmed.

It was noteworthy that 93.4% (226/242) of patients with pancreatic cancer were detected either by ApoAII-2+CIII-0 or by CA19-9 without compromising the specificity for healthy individuals [93.2% (110/118)]. Furthermore, ApoAII-2+CIII-0 was able to detect a broad spectrum of diseases in the pancreatic and hepatobiliary region with a sensitivity of >60% (Table 1). However, the reduction of ApoAII-2+CIII-0 did not seem to be specific to pancreatic and hepatobiliary diseases. Patients with esophageal, gastric, and colorectal cancers also showed reduction of plasma ApoAII-2+CIII-0 with a frequency of 36.4% (4/11), 23.9% (34/142), and 32.4% (46/142), respectively.

**Table 1:** ApoAII-2+ApoCIII-0 positivity rates in various gastrointestinal diseases (Cohort 4).

	*n*	ApoAII-2+ApoCIII-0 (<189.0 AU)	CA19-9(>37.0 U/ml )	ApoAII-2+ApoCIII-0 or CA19-9
**Healthy control**	118	2.54% (3/118)	5.08% (6/118)	6.78% (8/118)
**Invasive ductal adenocarcinoma** **of the pancreas**	243^a^	**65.8% (160/243)**	**79.8% (194/243)**	**93.4% (227/243)**
**Other malignant tumor of the pancreas**	18	**72.2% (13/18)**	22.2% (4/18)	**83.3% (15/18)**
**Benign tumor or cyst of the pancreas**	37	35.1% (13/37)	8.18% (3/37)	37.8% (14/37)
**Chronic pancreatitis**	14	**78.6% (11/14)**	14.3% (2/14)	**85.7% (12/14)**
**Hepatocellular carcinoma**	13	**69.2% (9/13)**	15.4% (2/13)	**69.2% (9/13)**
**Carcinoma of the duodenum**	10	**70.0%(7/10)**	30.0% (3/10)	**90.0% (9/10)**
**Gallbladder or cholangiocellular carcinoma**	44	56.8% (25/44)	**63.6% (28/44)**	**81.8% (36/44)**
**Benign disease of gallbladder or** **bile duct**	21	28.6% (6/21)	14.3% (3/21)	33.3% (7/21)
**Esophageal cancer**	11	36.4% (4/11)	18.2% (2/11)	36.4% (4/11)
**Gastric cancer**	142	23.9% (34/142)	16.2% (23/142)	33.1% (47/142)
**Colorectal cancer**	142	32.4% (46/142)	17.6% (25/142)	40.8% (58/142)
**Gastrointestinal stromal tumor**	3	0% (0/3)	0%(0/3)	0% (0/3)

a Includes one case of unknown clinical stage. Positivity rates higher than 60% are highlighted in boldface.

**Figure 5:**
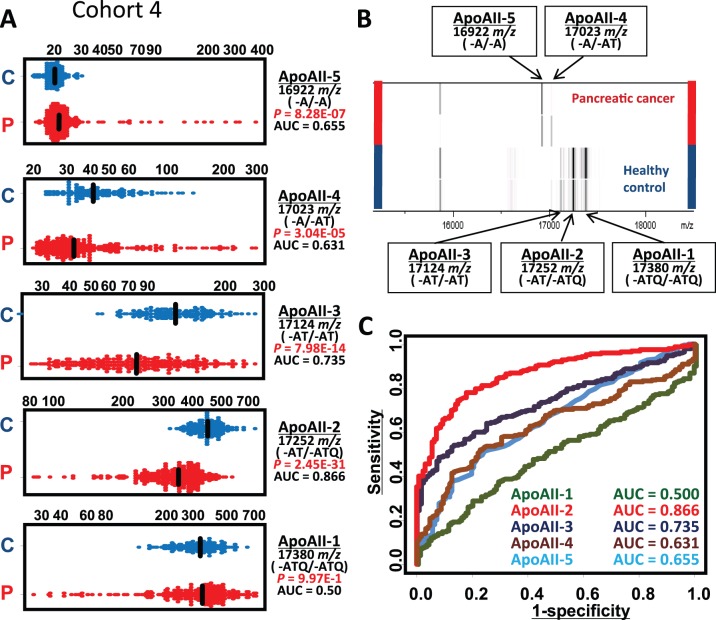
Differential modification of ApoAII. Distribution (peak intensity in arbitrary units) (**A**), spectra of representative cases (**B**), and ROC analysis (**C**) of the indicated modified forms of ApoAII in Cohort 4. C, healthy control (*n* = 128); P, pancreatic cancer (*n* = 249).

Similar significance was reproducibly obtained by MS measurements conducted independently at the National Cancer Center Research Institute (NCCRI) (Fig. 5 and data not shown).

### Novel Modification of ApoAII

The majority (>95%) of circulating ApoAII protein molecules form N-terminally disulfide-linked dimers. In addition to the 3 known isoforms of ApoAII (ApoAII-1 to -3) [14] we newly identified two other differently modified ApoAII dimers (ApoAII-4 and AII-5) (Figs. 3 and 5):

ApoAII-4 (17023 *m/z*), which lacks C-terminal glutamine (Q) and threonine (T) residues from one peptide and a glutamine residue from the other (-A/−AT).

ApoAII-5 (16922 *m/z*), which lacks C-terminal glutamine and threonine residues from both peptides (-A/−A).

ApoAII-1 (intact ApoAII with -ATQ/−ATQ termini) was not decreased in pancreatic cancer patients (Fig. 5A), indicating that the decrease of ApoAII-2 is not caused by a simple reduction of overall protein production, but by protein modification. The specific increase of ApoAII-5 in pancreatic cancer patients indicates that accelerated digestion may be one of the mechanisms responsible for the reduction of ApoAII-2 (see the Discussion section).

#### Production of antibody specific to ApoAII peptide with a -ATQ or -AT end

**Figure 6:**
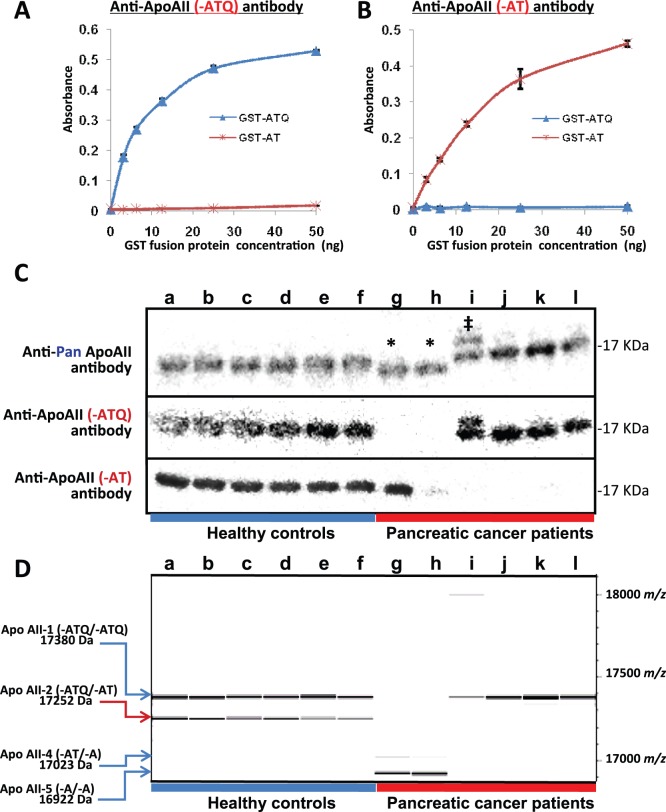
Detection of truncated ApoAII peptide. (**A and B**) Reactivity of anti-ApoAII (-ATQ) (**A**) and anti-ApoAII (-AT) (**B**) peptide antibodies with GST-tagged ApoAII monomers bearing -ATQ (GST-ATQ) (amino acids 1–100) and -AT (GST-AT) (amino acids 1–99) termini. (**C**) Non-denaturing immunoblot analysis of plasma samples from healthy controls (lanes a-f) and representative pancreatic cancer patients (lanes g-l) with antibodies against pan ApoAII protein (top), ApoAII (-ATQ) peptide (middle), and ApoAII (-ATQ) peptide (bottom). A pancreatic cancer patient (lane i) shows a slower-migrating ApoAII protein (indicated by ‡), but its molecular nature remains to be determined. (**D**) Gel-like image of oMALDI-QqTOF-MS spectra within the range 16,900–18,100 m/z for representative healthy controls (lanes a-f corresponding to **C**) and pancreatic cancer patients (lanes g-l corresponding to **C**).

To verify the truncation of ApoAII in patients with pancreatic diseases, we newly produced a polyclonal antibody that specifically recognizes the ApoAII peptide with an intact C-terminus (-ATQ), but not that with a cleaved C-terminus (-AT) (Fig. 6A), as well as a polyclonal antibody that specifically recognizes the Apo-AII peptide with a cleaved (-AT) C-terminus, but not that with an intact (-ATQ) C-terminus (Fig. 6B).

The results of immunoblot analyses using these antibodies (Fig. 6C) were consistent with those obtained by oMALDI-QqTOF-MS (Fig. 6D). In 2 pancreatic cancer patients (lanes g and h) who lacked ApoAII-1 and ApoAII-2 but possessed ApoAII-4 and ApoAII-5 (Fig. 6D), immunoblot analysis under non-denaturing (native) conditions revealed loss of the 17-kDa ApoAII peptide detectable with anti-ApoAII (-ATQ) antibody (middle, Fig. 6C), and a faster-migrating (smaller) ApoAII peptide was detected with anti-Pan Apo-AII antibody (top, indicated by asterisks, Fig. 6C).

## Discussion

Pancreatic cancer is a devastating disease, but a substantial proportion of patients can be cured if detection is possible at an early stage [20], [21]. To improve the outcome of pancreatic cancer, establishment of an effective screening method is essential. Although the decrease of the modified plasma/serum apolipoproteins reported here is not specific to pancreatic cancer, this test is minimally invasive and in this sense might be considered for application to primary screening of asymptomatic patients with pancreatic cancer as well as other gastrointestinal diseases among the general (apparently healthy) population, if followed by an appropriate second screening for differential diagnosis, such as enhanced CT or ultrasonography [22]. Screening of blood by MS would provide asymptomatic patients with a precious chance to receive clinical attention and eventually increase the rate of discovery of early-stage pancreatic cancer. However, the sensitivity and specificity of the current set (ApoAII-2+CIII-0), even when combined with CA19-9, does not seem to be sufficient, and the number of patients with early-stage pancreatic cancer examined in this study was small. It will be necessary to conduct a prospective randomized trial before definite conclusions can be drawn about the utility of reduced ApoAII-2 and ApoCIII-0 in the setting of cancer screening.

Most tumor markers secreted by cancer cells into the circulation do not show a significant increase when cancer is in its early stage and the tumor burden relative to the entire body is small. This nature of most conventional tumor markers makes their application to cancer screening difficult [3], [23]. However, unlike conventional tumor markers, the modified apolipoproteins were reduced in patients with pancreatic cancer and other gastrointestinal diseases. Apolipoproteins are produced mainly in the liver, and ApoAII and ApoCIII proteins are unlikely to be produced by cancer cells. However, 17252-*m/z* ApoAII heterodimer with -AT/−ATQ C-terminal ends (ApoAII-2) and 8766-*m/z* unglycosylated ApoCIII (ApoCIII-0) protein were found to be decreased most significantly in patients with pancreatic cancer, including those with early-stage (stages I and II) disease. These findings indicate that the reduction of these specific forms of apolipoprotein is not a consequence of reduced protein production due to destruction of pancreatic tissue, but is attributable to certain post-translational mechanisms.

It has been reported that increased serum carboxyl- and aminopeptidase activities generate cancer-specific peptidome patterns through cleavage of the C- or N-termini of various plasma/serum proteins [24]. Increased serum carboxypeptidase A activity has been reported in patients with early-stage pancreatic cancer [25]. Synthetic *N*-acetyl-phenyl-L-3-thiaphenylamine has been used as a sensitive substrate to measure the activity of the enzyme, but the small initial cleavage of a C-terminal glutamine (Q) residue may prime/sensitize ApoAII protein to further proteolytic digestion, and thus the degradation/decrease of ApoAII-2 might be detectable sensitively even in patients with early-stage pancreatic cancer. The reciprocal increase of the ApoAII homodimer with -A/−A ends (ApoAII-5) supports this notion (Fig. 5A). Some pancreatic cancer patients even showed complete depletion of ApoAII-1 and -2 proteins and the appearance of novel ApoAII-4 and -5 proteins (lanes f and g, Fig. 6C and 6D), indicating that enhancement of proteolytic digestion is responsible for the reduction of ApoAII-2 in these patients.

There is a weakness to be overcome before the current findings can be applied clinically. It is generally accepted that a decrease in the level of any biomarker is difficult to employ as a diagnostic measure, because a highly reproducible measurement system is needed in order to avoid false negativity. The small protein modifications reported here can currently be detected and quantified only by MS. We constructed a robust analytical system employing automated sample preparation using magnetic beads [26]. This automation reduced fluctuations in handling, and the use of orthogonal MS eradicated any mass inaccuracy caused by the slightly uneven surfaces of MALDI plates. We also carefully eliminated any possible pre-analytical bias of sampling procedures and avoided using complicated computing algorithms. However, MS-based protein quantification is still a complicated technology for routine clinical use, and its application to cancer screening requires further improvement of robustness, throughput, and cost efficiency.

In this study we successfully produced a pair of antibodies specifically recognizing the intact ApoAII peptide with an -ATQ terminus and the truncated ApoAII peptide with a -AT terminus (Fig. 6). We are now in the process of establishing a sandwich enzyme-linked immunosorbent assay to quantify the ApoAII-2 heterodimer with a hemi-truncated C-terminus (-ATQ/−AT) using these antibodies. We think that the perfection of this assay will greatly facilitate the clinical applicability of the present findings.

## Supporting Information

Figure S1 Decrease of the 17252-m/z peak in patients with pancreatic cancer.
(PDF)

Figure S2 Identification of ApoAII-2 heterodimer.
(PDF)

Figure S3 Identification of ApoAII-2 heterodimer.
(PDF)

Figure S4 Confirmation of protein identity by immunoprecipitation and MS.
(PDF)

Figure S5 Identification of ApoCIII.
(PDF)

Figure S6 Correlation of ApoAII-2 and ApoCIII0.
(PDF)

Table S1 Clinical characteristics of individuals in Cohorts 1 to 3.
(PDF)

Table S2 Clinical characteristics of individuals in Cohort 4 (n = 833).
(PDF)

Table S3 Reduction of plasma ApoAII-2 and ApoCIII-0 in patients with pancreatic cancer (Cohorts 1 and 2).
(PDF)

Table S4 Validation of ApoAII-2 and ApoCIII-0 in the German cohort (Cohort 3).
(PDF)

Table S5 Reduction of ApoAII-2+ApoCIII-0 in patients with early-stage pancreatic cancer (Cohort 4).
(PDF)

Methods S1 (PDF)
