# Supplementary material for: Altered Plasma Apolipoprotein Modifications in Patients with Pancreatic Cancer: Protein Characterization and Multi-Institutional Validation
Source: PLoS One. 2012 Oct 8;7(10):e46908. doi: 10.1371/journal.pone.0046908 (PMC3466211; doi:10.1371/journal.pone.0046908)
Supplement: Figure S1 — Decrease of the 17252-m/z peak in patients with pancreatic cancer. (PDF) [file pone.0046908.s001.pdf]

Supplementary Figure S1

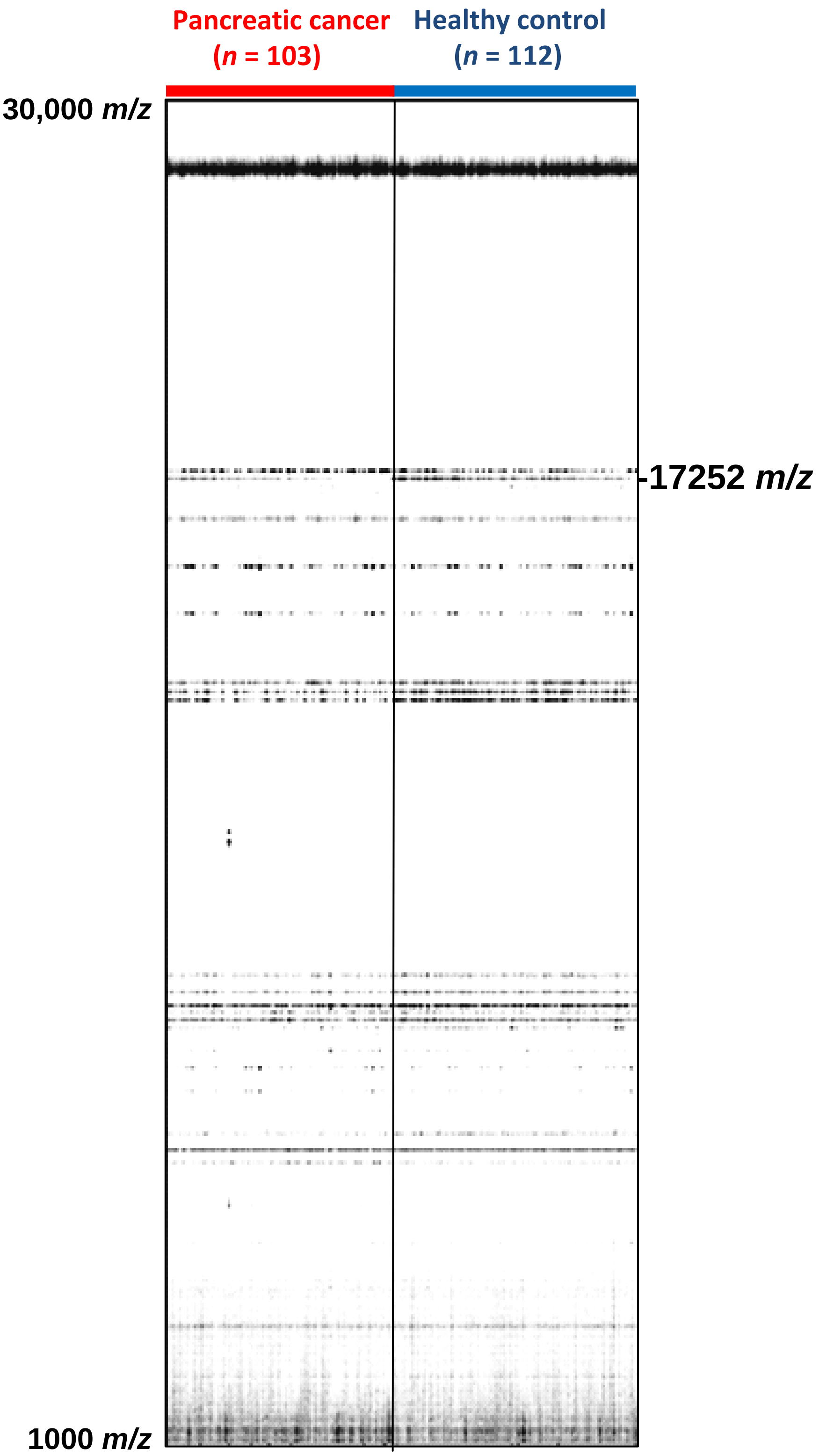

Supplementary Figure S1. Decrease of the 17252-m/z peak in patients with pancreatic cancer.

Gel-like view converted from the entire MS spectra (1000 to 30,000 m/z) of 215 cases in Cohort 1. Plasma samples were randomized prior to MS analysis and rearranged for presentation.
