# Supplementary material for: Altered Plasma Apolipoprotein Modifications in Patients with Pancreatic Cancer: Protein Characterization and Multi-Institutional Validation
Source: PLoS One. 2012 Oct 8;7(10):e46908. doi: 10.1371/journal.pone.0046908 (PMC3466211; doi:10.1371/journal.pone.0046908)
Supplement: Figure S2 — Identification of ApoAII-2 heterodimer. (PDF) [file pone.0046908.s002.pdf]

Supplementary Figure S2

ApoAII fragment with –AT end (1193.1220 m/z) (*Z* = 2)

KAGTELVNFLSYFVELGTQPAT

Molecular weight of precursor
Experimental molecular weight: 2384.2294 m/z
Calculated molecular weight: 2384.2264 m/z
Error rate between experimental MS and calculated MS : 1.26 ppm

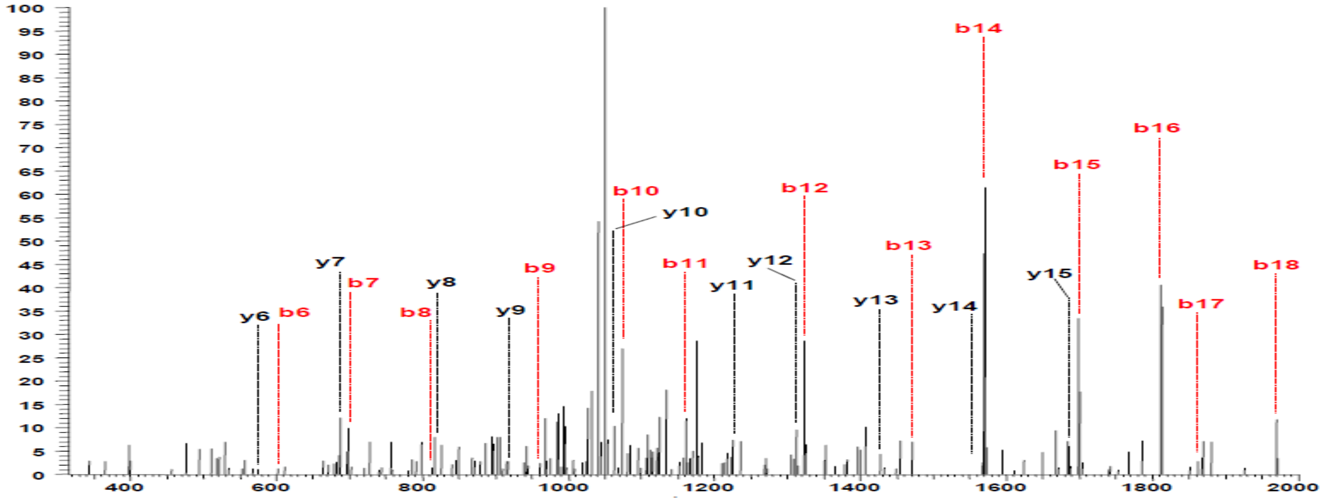

#	b	b⁺⁺	b*	b*⁺⁺	b⁰	b⁰⁺⁺	Seq.	y	y⁺⁺	y*	y*⁺⁺	y⁰	y⁰⁺⁺	#
1	129.10	65.05	112.08	56.54			K							22
2	200.14	100.57	183.11	92.06			A	2257.14	1129.07	2240.11	1120.56	2239.13	1120.07	21
3	257.16	129.08	240.13	120.57			G	2186.10	1093.55	2169.08	1085.04	2168.09	1084.55	20
4	358.21	179.61	341.18	171.09	340.20	170.60	T	2129.08	1065.04	2112.05	1056.53	2111.07	1056.04	19
5	487.25	244.13	470.22	235.62	469.24	235.12	E	2028.03	1014.52	2011.01	1006.01	2010.02	1005.51	18
6	600.34	300.67	583.31	292.16	582.32	291.67	L	1898.99	950.00	1881.96	941.49	1880.98	940.99	17
7	699.40	350.21	682.38	341.69	681.39	341.20	V	1785.91	893.46	1768.88	884.94	1767.90	884.45	16
8	813.45	407.23	796.42	398.71	795.44	398.22	N	1686.84	843.92	1669.81	835.41	1668.83	834.92	15
9	960.51	480.76	943.49	472.25	942.50	471.76	F	1572.79	786.90	1555.77	778.39	1554.78	777.90	14
10	1073.60	537.30	1056.57	528.79	1055.59	528.30	L	1425.73	713.37	1408.70	704.85	1407.72	704.36	13
11	1160.63	580.82	1143.60	572.31	1142.62	571.81	S	1312.64	656.82	1295.62	648.31	1294.63	647.82	12
12	1323.69	662.35	1306.67	653.84	1305.68	653.35	Y	1225.61	613.31	1208.58	604.80	1207.60	604.30	11
13	1470.76	735.89	1453.74	727.37	1452.75	726.88	F	1062.55	531.78	1045.52	523.26	1044.54	522.77	10
14	1569.83	785.42	1552.80	776.91	1551.82	776.41	V	915.48	458.24	898.45	449.73	897.47	449.24	9
15	1698.87	849.94	1681.85	841.43	1680.86	840.94	E	816.41	408.71	799.38	400.20	798.40	399.70	8
16	1811.96	906.48	1794.93	897.97	1793.95	897.48	L	687.37	344.19	670.34	335.67	669.36	335.18	7
17	1868.98	934.99	1851.95	926.48	1850.97	925.99	G	574.28	287.65	557.26	279.13	556.27	278.64	6
18	1970.03	985.52	1953.00	977.00	1952.02	976.51	T	517.26	259.13	500.24	250.62	499.25	250.13	5
19	2098.09	1049.55	2081.06	1041.03	2080.07	1040.54	Q	416.21	208.61	399.19	200.10	398.20	199.61	4
20	2195.14	1098.07	2178.11	1089.56	2177.13	1089.07	P	288.16	144.58			270.14	135.58	3
21	2266.18	1133.59	2249.15	1125.08	2248.16	1124.59	A	191.10	96.05			173.09	87.05	2
22							T	120.07	60.54			102.05	51.53	1

Supplementary Figures S2 and S3. Identification of ApoAII-2 heterodimer. Labeled MS/MS spectra of the 17252-m/z peak, which contained two peptide fragments (1193.1220 *m/z* and 1257.1500 *m/z*) with different C-terminal ends [-AT (Fig. S2) and -ATQ (Fig. S3)]. MS/MS was performed using ESI-LTQ Orbitrap XL, and the peak list was generated by Mascot software. The theoretical precursor MS (doubly charged) of KAGTELVNFLSYFVELGTQPATQ was 2512.2850 Da, and the observed MS was 2512.2854 Da. The difference was 0.16 ppm. The theoretical precursor MS (doubly charged) of KAGTELVNFLSYFVELGTQPAT was 2384.2294 Da, and the observed MS was 2384.2264 Da. The difference was 1.26 ppm.
