# Supplementary material for: Altered Plasma Apolipoprotein Modifications in Patients with Pancreatic Cancer: Protein Characterization and Multi-Institutional Validation
Source: PLoS One. 2012 Oct 8;7(10):e46908. doi: 10.1371/journal.pone.0046908 (PMC3466211; doi:10.1371/journal.pone.0046908)
Supplement: Figure S4 — Confirmation of protein identity by immunoprecipitation and MS. (PDF) [file pone.0046908.s004.pdf]

Supplementary Figure S4

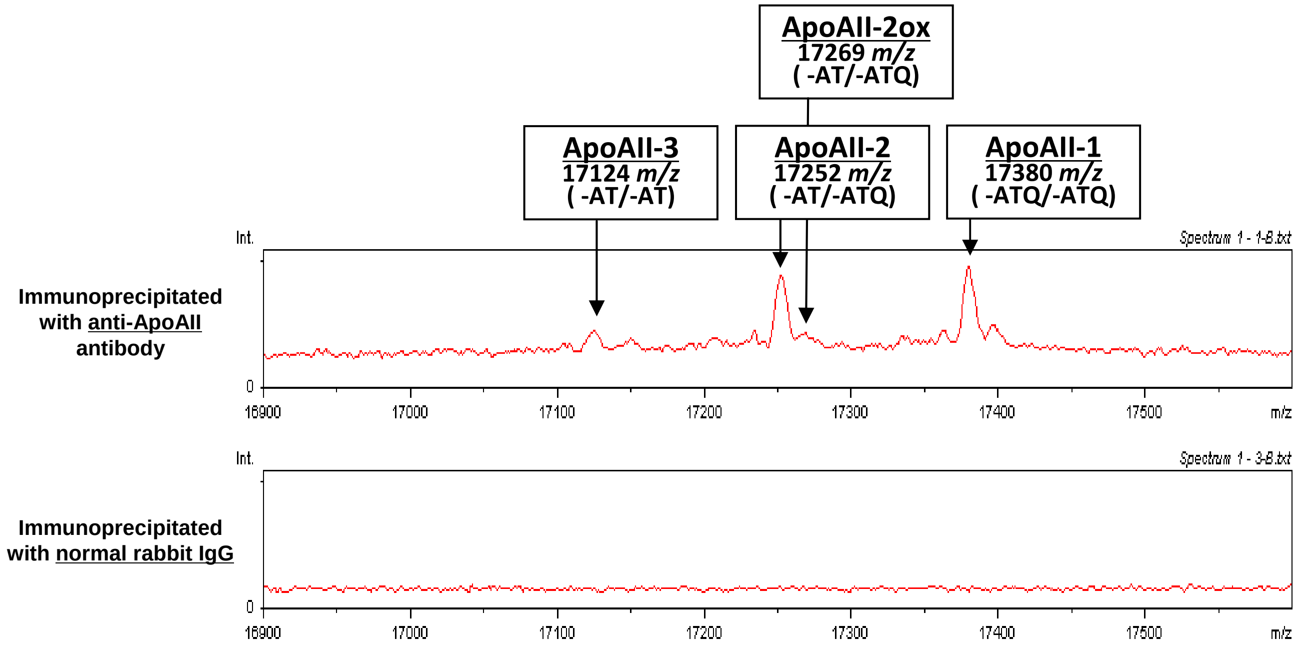

Supplementary Figure S4. Confirmation of protein identity by immunoprecipitation and MS.
A plasma mixture from healthy volunteers was immunoprecipitaed with anti-ApoAII antibody or normal rabbit IgG (negative control), and the precipitated proteins were analyzed by oMALDI-QqTOF-MS.
