# Supplementary material for: Altered Plasma Apolipoprotein Modifications in Patients with Pancreatic Cancer: Protein Characterization and Multi-Institutional Validation
Source: PLoS One. 2012 Oct 8;7(10):e46908. doi: 10.1371/journal.pone.0046908 (PMC3466211; doi:10.1371/journal.pone.0046908)
Supplement: Figure S5 — Identification of ApoCIII. (PDF) [file pone.0046908.s005.pdf]

Supplementary Figure S5

TAKDALSSVQESQVAQQAR
1009.0897 *m/z* (*Z* = 2)

Molecular weight of precursor
Experimental molecular weight: 2016.1648 m/z
Calculated molecular weight: 2016.0236 m/z
Error rate between experimental MS and calculated MS : 70.0 ppm

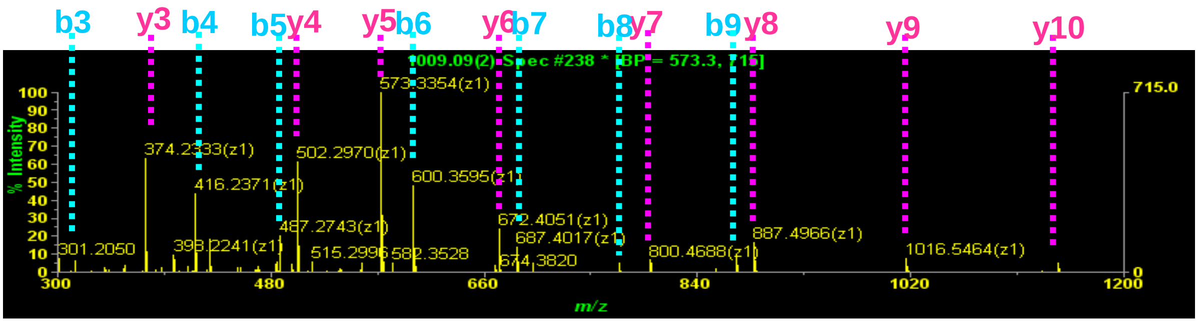

#	b	b^{++}	b^*	b^{*++}	b^0	b^{0++}	Seq.	y	y^{++}	y^*	y^{*++}	y^0	y^{0++}	#
1	102.0550	51.5311			84.0444	42.5258	T							19
2	173.0921	87.0497			155.0815	78.0444	A	1915.9832	958.4952	1898.9566	949.9820	1897.9726	949.4900	18
3	301.1870	151.0972	284.1605	142.5839	283.1765	142.0919	K	1844.9461	922.9767	1827.9195	914.4634	1826.9355	913.9714	17
4	416.2140	208.6106	399.1874	200.0974	398.2034	199.6053	D	1716.8511	858.9292	1699.8246	850.4159	1698.8406	849.9239	16
5	487.2511	244.1292	470.2245	235.6159	469.2405	235.1239	A	1601.8242	801.4157	1584.7976	792.9025	1583.8136	792.4104	15
6	600.3352	300.6712	583.3086	292.1579	582.3246	291.6659	L	1530.7871	765.8972	1513.7605	757.3839	1512.7765	756.8919	14
7	687.3672	344.1872	670.3406	335.6740	669.3566	335.1819	S	1417.7030	709.3551	1400.6764	700.8419	1399.6924	700.3499	13
8	774.3992	387.7032	757.3727	379.1900	756.3886	378.6980	S	1330.6710	665.8391	1313.6444	657.3258	1312.6604	656.8338	12
9	873.4676	437.2374	856.4411	428.7242	855.4571	428.2322	V	1243.6389	622.3231	1226.6124	613.8098	1225.6284	613.3178	11
10	1001.5262	501.2667	984.4997	492.7535	983.5156	492.2615	Q	1144.5705	572.7889	1127.5440	564.2756	1126.5600	563.7836	10
11	1130.5688	565.7880	1113.5422	557.2748	1112.5582	556.7828	E	1016.5119	508.7596	999.4854	500.2463	998.5014	499.7543	9
12	1217.6008	609.3040	1200.5743	600.7908	1199.5903	600.2988	S	887.4694	444.2383	870.4428	435.7250	869.4588	435.2330	8
13	1345.6594	673.3333	1328.6329	664.8201	1327.6488	664.3281	Q	800.4373	400.7223	783.4108	392.2090			7
14	1444.7278	722.8675	1427.7013	714.3543	1426.7172	713.8623	V	672.3788	336.6930	655.3522	328.1797			6
15	1515.7649	758.3861	1498.7384	749.8728	1497.7544	749.3808	A	573.3103	287.1588	556.2838	278.6455			5
16	1643.8235	822.4154	1626.7970	813.9021	1625.8129	813.4101	Q	502.2732	251.6402	485.2467	243.1270			4
17	1771.8821	886.4447	1754.8555	877.9314	1753.8715	877.4394	Q	374.2146	187.6110	357.1881	179.0977			3
18	1842.9192	921.9632	1825.8926	913.4500	1824.9086	912.9580	A	246.1561	123.5817	229.1295	115.0684			2
19							R	175.1190	88.0631	158.0924	79.5498			1

Supplementary Figure S5. Identification of ApoCIII.
Labeled MS/MS spectrum of a peptide fragment derived from the 8766-m/z peak. The spectrum was obtained by LIT-qTOF-MS.
