# Supplementary material for: Altered Plasma Apolipoprotein Modifications in Patients with Pancreatic Cancer: Protein Characterization and Multi-Institutional Validation
Source: PLoS One. 2012 Oct 8;7(10):e46908. doi: 10.1371/journal.pone.0046908 (PMC3466211; doi:10.1371/journal.pone.0046908)
Supplement: Figure S6 — Correlation of ApoAII-2 and ApoCIII0. (PDF) [file pone.0046908.s006.pdf]

Supplementary Figure S6

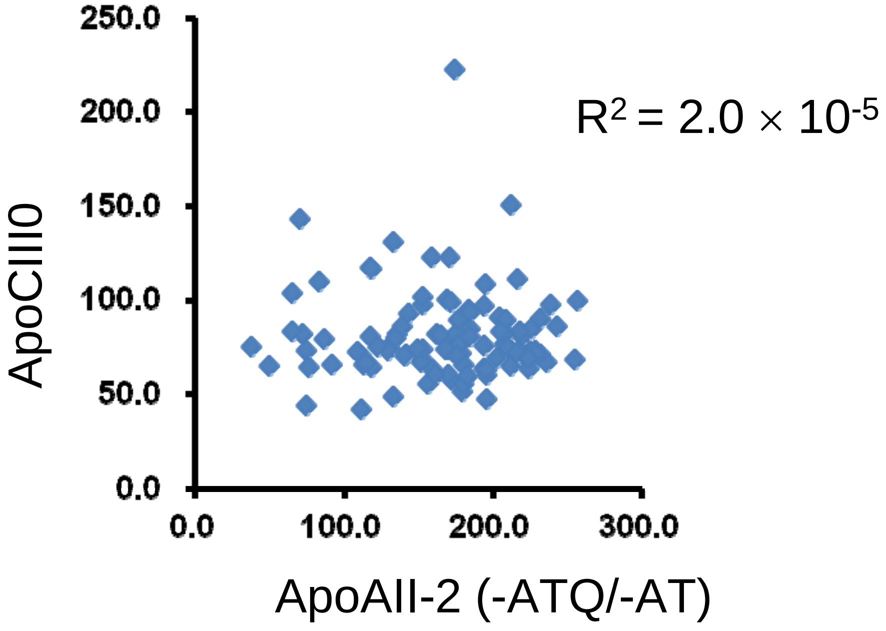

Supplementary Figure S6. Correlation of ApoAII-2 and ApoCIII0.
Distribution of 17,252-m/z (ApoAII-2) and 8766-m/z (ApoCIII0) peaks for 112 pancreatic cancer patients in Cohort 1. There was no significant correlation between the levels of the 2 proteins [Coefficient of determination (R2) = 2.0×10^{-5}].
