# Supplementary material for: Altered Plasma Apolipoprotein Modifications in Patients with Pancreatic Cancer: Protein Characterization and Multi-Institutional Validation
Source: PLoS One. 2012 Oct 8;7(10):e46908. doi: 10.1371/journal.pone.0046908 (PMC3466211; doi:10.1371/journal.pone.0046908)
Supplement: Table S1 — Clinical characteristics of individuals in Cohorts 1 to 3. (PDF) [file pone.0046908.s007.pdf]

Supplementary Table S1. Clinical characteristics of individuals in Cohorts 1 to 3

Collection period	Cohort 1 (*n* = 215) (2002-2005)			Cohort 2 (*n* = 103) (2003-2005)			Cohort 3 (*n* = 163) (2003-2006)				
	**Healthy control**	**Pancreatic cancer**	***P*-value**	**Healthy control**	**Pancreatic cancer**	***P*-value**	**Healthy control**	**Pancreatic cancer**	***P*-value**	**Chronic pancreatitis**	***P*-value**
**Number of cases**	112	103		41	62		53	52		58	
**Age (mean ± SD)**	61.3 ± 13.0	61.5 ± 9.67	0.8795[a]	61.5 ± 11.2	63.3 ± 8.34	0.3729[a]	39.1 ± 15.6	63.1 ± 9.85	4.67E-15[a]	50.3 ± 8.93	9.08E-6[a]
**Gender**			0.7134[b]			0.5996[b]			0.7785[b]		0.0807[b]
Male	57	55		23	38		31	29		43	
Female	55	48		18	24		22	23		15	

[a]Student *t*-test

[b]χ2 test
