# Supplementary material for: Altered Plasma Apolipoprotein Modifications in Patients with Pancreatic Cancer: Protein Characterization and Multi-Institutional Validation
Source: PLoS One. 2012 Oct 8;7(10):e46908. doi: 10.1371/journal.pone.0046908 (PMC3466211; doi:10.1371/journal.pone.0046908)
Supplement: Table S2 — Clinical characteristics of individuals in Cohort 4 (n = 833). (PDF) [file pone.0046908.s008.pdf]

Supplementary Table S2. Clinical characteristics of individuals in Cohort 4 (*n* = 833)

Disease		Age		Gender		
		Mean ± SD	*P*-value[a]	Male	Female	*P*-value[b]
Healthy	*n* = 128	46.6 ± 16.8		83	45	
Invasive ductal adenocarcinoma of the pancreas	*n* = 249	64.4 ± 9.11	5.45E-22	146	103	0.221
Other malignat tumor of the pancreas	*n* = 18	68.3 ± 9.74	3.60E-09	12	6	0.897
Benign tumor or cyst of the pancreas	*n* = 38	63.5 ± 11.0	1.17E-10	17	21	0.0239
Chronic pancreatitis	*n* = 14	60.2 ± 10.2	2.54E-04	12	2	0.119
Hepatocellular carcinoma	*n* = 13	69.2 ± 7.47	2.02E-09	11	2	0.154
Carcinoma of the duodenum	*n* = 10	69.8 ± 7.87	4.77E-07	5	5	0.337
Gallbladdar or cholangiocellular carcinoma	*n* = 44	68.0 ± 9.91	3.73E-18	27	17	0.654
Benign disease of the gallbladder or bile duct	*n* = 21	56.0 ± 18.1	3.54E-02	8	13	0.0183
Esophageal cancer	*n* = 11	64.1 ± 9.20	4.01E-05	10	1	0.0804
Gastric cancer	*n* = 142	65.9 ± 10.7	6.22E-23	105	37	0.114
Colorectal cancer	*n* = 142	64.0 ± 11.1	2.44E-19	82	60	0.214
Gastrointestinal stromal tumor	*n* = 3	68.3 ± 3.79	9.21E-04	3	0	0.208

[a]Student *t*-test

[b]$\chi 2$ test
