# Supplementary material for: Altered Plasma Apolipoprotein Modifications in Patients with Pancreatic Cancer: Protein Characterization and Multi-Institutional Validation
Source: PLoS One. 2012 Oct 8;7(10):e46908. doi: 10.1371/journal.pone.0046908 (PMC3466211; doi:10.1371/journal.pone.0046908)
Supplement: Table S3 — Reduction of plasma ApoAII-2 and ApoCIII-0 in patients with pancreatic cancer (Cohorts 1 and 2). (PDF) [file pone.0046908.s009.pdf]

Supplementary Table S3. Reduction of plasma ApoAII-2 and ApoCIII-0 in patients with pancreatic cancer (Cohorts 1 and 2)

	Cohort 1 (*n* = 215)					Cohort 2 (*n* = 103)				
	**Healthy control** Mean ± SD (*n* = 112)	**Pancreatic cancer** Mean ± SD (*n* = 103)	*P* -value[a]	AUC	95% CI[b]	**Healthy control** Mean ± SD (*n* = 41)	**Pancreatic cancer** Mean ± SD (*n* = 62)	*P* -value[a]	AUC	95% CI[b]
ApoAII-3	95.5 ± 32.4	62.4 ± 32.6	1.48E-12	0.780	(0.716-0.843)	98.1 ± 30.1	58.1 ± 34.2	8.23E-09	0.837	(0.758-0.961)
**ApoAII-2**	235.3 ± 38.0	165.2 ± 49.8	1.36E-21	**0.877**	(0.832-0.922)	237.5 ± 44.2	157.9 ± 54.8	9.72E-11	**0.878**	(0.812-0.944)
**ApoAII-2ox**	79.0 ± 9.8	63.6 ± 11.1	5.14E-20	**0.862**	(0.812-0.911)	80.1 ± 11.4	61.9 ± 13.5	2.43E-09	**0.849**	(0.774-0.923)
ApoAII-1	212.6 ± 73.0	255.3 ± 116.5	3.55E-03	0.615	(0.537-0.963)	207.5 ± 69.9	244.3 ± 100.3	1.21E-02	0.647	(0.539-0.755)
**ApoCIII-0**	105.6 ± 24.1	80.9 ± 24.1	4.35E-14	**0.798**	(0.738-0.859)	112.0 ± 27.9	72.7 ± 22.5	1.31E-11	**0.895**	(0.837-0.954)
ApoCIII-1	169.0 ± 29.2	147.9 ± 30.9	1.37E-06	0.691	(0.620-0.761)	180.8 ± 31.4	139.4 ± 39.3	1.21E-07	0.809	(0.728-0.890)
ApoCIII-2	56.1 ± 7.16	55.9 ± 8.78	6.43E-01	0.518	(0.441-0.596)	59.3 ± 7.31	54.5 ± 9.72	9.58E-03	0.651	(0.547-0.756)
ApoAI	177.2 ± 21.3	166.7 ± 27.2	9.10E-03	0.603	(0.528-0.679)	191.7 ± 27.6	173.3 ± 36.3	1.76E-02	0.639	(0.531-0.747)
**ApoAII-2+CIII-0**	340.9 ± 49.3	246.0 ± 55.5	1.83E-24	**0.903**	(0.863-0.944)	349.5 ± 57.7	230.6 ± 68.3	2.88E-13	**0.926**	(0.876-0.977)

[a]Calculated by Mann-Whitney U-test. AUC values larger than 0.8 are highlighted in boldface.
