# Supplementary material for: Altered Plasma Apolipoprotein Modifications in Patients with Pancreatic Cancer: Protein Characterization and Multi-Institutional Validation
Source: PLoS One. 2012 Oct 8;7(10):e46908. doi: 10.1371/journal.pone.0046908 (PMC3466211; doi:10.1371/journal.pone.0046908)
Supplement: Table S4 — Validation of ApoAII-2 and ApoCIII-0 in the German cohort (Cohort 3). (PDF) [file pone.0046908.s010.pdf]

Supplementary Table S4. Validation of ApoAII-2 and ApoCIII-0 in the German cohort (Cohort 3)

	Healthy control (*n* = 53)	Pancreatic cancer (*n* = 52)				Chronic pancreatitis (*n* = 58)			
	Mean ± SD	Mean ± SD	*P* -value[a]	AUC	95% CI[b]	Mean ± SD	*P* -value[a]	AUC	95% CI[b]
**ApoAII-2**	278.2 ± 41.6	128.3 ± 72.8	6.21E-16	**0.958**	(0.920-0.996)	148.3 ± 100.0	5.74E-11	**0.861**	(0.790-0.932)
**ApoCIII-0**	79.5 ± 21.0	64.6 ± 18.2	7.57E-05	0.724	(0.626-0.823)	61.8 ± 22.3	3.63E-05	0.728	(0.633-0.822)
**ApoAII-2+CIII-0**	357.6 ± 54.0	192.9 ± 82.6	3.63E-15	**0.946**	(0.901-0.990)	210.1 ± 116.5	8.86E-11	**0.858**	(0.787-0.928)

[a]Calculated by Mann-Whitney U-test. AUC values larger than 0.8 are highlighted in boldface.
