# Supplementary material for: Altered Plasma Apolipoprotein Modifications in Patients with Pancreatic Cancer: Protein Characterization and Multi-Institutional Validation
Source: PLoS One. 2012 Oct 8;7(10):e46908. doi: 10.1371/journal.pone.0046908 (PMC3466211; doi:10.1371/journal.pone.0046908)
Supplement: Table S5 — Reduction of ApoAII-2+ApoCIII-0 in patients with early-stage pancreatic cancer (Cohort 4). (PDF) [file pone.0046908.s011.pdf]

Supplementary Table S5. Reduction of ApoAII-2+ApoCIII-0 in patients with early-stage pancreatic cancer (Cohort 4)

		ApoAII-2+CIII-0						CA19-9						ApoAII-2+CIII-0 and CA19-9	
		Mean ± SD	*P*-value[a]	AUC	95% CI[b]	Sensitivity	Specificity	Mean ± SD	*P*-value[a]	AUC	95% CI[b]	Sensitivity	Specificity	Sensitivity	Specificity
Healthy	*n* = 118	269.0 ± 54.4				-	97.50	11.8 ± 11.6				-	94.92	-	93.22
All stages	*n* = 242	167.9 ± 52.9	4.25E-38	0.919	(0.891-0.945)	66.12	97.50	2283.4 ± 3444.9	2.72E-36	0.908	(0.875-0.938)	79.75	94.92	93.39	93.22
Stage I	*n* = 7	180.5 ± 72.5	1.12E-03	0.868	(0.658-1.000)	71.43	97.50	279.5 ± 457.8	1.00E-02	0.790	(0.599-0.939)	57.14	94.92	100.00	93.22
Stage II	*n* = 35	165.5 ± 49.9	1.04E-14	0.931	(0.885-0.978)	68.57	97.50	554.6 ± 987.2	1.89E-15	0.943	(0.897-0.979)	71.43	94.92	97.14	93.22
Stage III	*n* = 69	167.3 ± 54.2	1.96E-21	0.917	(0.875-0.959)	66.67	97.50	1108.8 ± 2039.6	1.07E-22	0.930	(0.878-0.974)	84.06	94.92	94.20	93.22
Stage IV	*n* = 131	167.9 ± 52.4	3.40E-30	0.919	(0.886-0.952)	64.89	97.50	3471.0 ± 4047.8	7.02E-27	0.894	(0.844-0.939)	80.92	94.92	91.60	93.22

[a]Mann-Whitney *U*-test
