# Supplementary material for: Altered Plasma Apolipoprotein Modifications in Patients with Pancreatic Cancer: Protein Characterization and Multi-Institutional Validation
Source: PLoS One. 2012 Oct 8;7(10):e46908. doi: 10.1371/journal.pone.0046908 (PMC3466211; doi:10.1371/journal.pone.0046908)
Supplement: Methods S1 — (PDF) [file pone.0046908.s012.pdf]

Supplementary Methods

Robotic chromatography

Samples (50 µl) were desalted/concentrated in quadruplicate by chromatography over hydrophobic C8-coated magnetic beads (Bruker Daltonics, Bremen, Germany) (at the NCCRI) [1] or originally manufactured equivalent beads (at Molecuence). Several lots of beads were mixed to make large stocks to avoid lot-to-lot variations. After washing, proteins attached to the beads were eluted by addition of 10 µl of 80% isopropanol (Wako, Osaka, Japan). The eluent was mixed (1:10, v/v) with α-cyano-4-hydroxycinnamic acid saturated in 0.1% trifluoroacetic acid/50% acetonitrile/H_2O (Wako). Programmed chromatography was performed using a ClinProt Robot GA (Bruker Daltonics) (at the NCCRI) or a Biomek FX Laboratory Automation Robot (Beckman Coulter, Fullerton, CA) (at Molecuence) [1]. Two aliquots of a mixture of volunteer plasma were processed simultaneously with every 22 test samples to ensure chromatographic consistency.

Quantitative MS

The quadruplicate desalted/concentrated samples (0.8 µl/spot) for each case were spotted in quadruplicate on 16 random positions of disposable 384-well MALDI plates [$(2 + 22) \times 4 \times 4 = 384$] (PerkinElmer) and dried. Quantitative MS data for plasma/serum proteins were obtained using oMALDI-QqTOF-MS (prOTOF 2000; PerkinElmer, Boston, MA) at the following settings: Laser shot, 180; Total time, 3 min 13 s; Laser energy, 79%; Laser frequency, 100 Hz; declustering, 32.0 V; Cooling flow, 170.0 ml/min; Mass range, 800-30,000 Da; Expected high mass, 31,000 Da; Focusing flow, 202 ml/min; Laser pattern, ring. MS peaks were detected, normalized, and quantified using the in-house ProteoJudge software package [Bin size, 4; Smoothing parameter (Gaussian width, 3; Sigma, 1)； Normalization (Width, 400; Level, 0.1; Total intensity, 5,300,000; Minimum m/z, 2000; Maximum m/z, 30,000); Peak detection (Range, 1000-30,000 m/z; Minimum intensity, 3; Minimum S/N, 10; Alignment m/z, 0.015%; Detection ratio, 15%)] and visualized using Mass Navigator software (Mitsui Knowledge Industry, Tokyo, Japan) [2]. MS accuracy was calibrated externally with a peptide molecular mass standard (proXPRESSION kit, PerkinElmer).

Tandem mass spectrometry (MS/MS)

A 40-µl plasma sample from a healthy volunteer was diluted 10-fold with 0.1% trifluoroacetic acid and separated on a gradient of 20-80% acetonitrile/H_2O using high-performance liquid chromatography (HPLC) (AKTA explore 10S, GE Healthcare,

Buckinghamshire, UK) equipped with a 3-µm id, 4.6×50-mm C8 reverse-phase column (Unison UK-C8, Imtakt, Kyoto, Japan) at a flow rate of 1 ml/min over 33.2 min. Each fraction was monitored by oMALDI-QqTOF-MS, and the 17,252-m/z protein was found to appear in the 53-54% acetonitrile fraction. This fraction was digested with sequence-grade modified trypsin (Promega, Madison, WI) for 16 h, separated by HPLC (HTS/HTC PAL, CTC Analytics, Zwingen, Switzerland) equipped with a 3-µm id, 0.2×50-mm C18 column (Magic C18AQ, Michrom Bioresources, Auburn, CA), and analyzed by LTQ Orbitrap XL (Thermo Fisher Scientific, Waltham, MA).

The MS/MS data were searched against UniProtKB/Swiss-Prot release 57.9 (Oct. 13, 2009) using Mascot Daemon (version 2.2.2) (Matrix Science, London, UK). The search parameters used were as follows: Database, human proteins; Enzyme, trypsin; Maximum missed cleavage, 2; Peptide charge; 1+, 2+, 3+; Peptide tolerance, 10 ppm; MS/MS tolerance, 0.8 Da; Mass; monoisotopic; Fixed modification, carbamidomethyl (C); Variable modification, Oxidation (M).

MS/MS spectra were obtained from the 8766 *m/z* protein using linear ion trap (LIT)-qTOF-MS (NanoFrontier eLD, Hitachi High-Technology Corporation, Tokyo, Japan). Peak lists were generated using NanoFrontier LD Data Processing (version 3807211-01) (Hitachi High-Technology Corporation) and searched against the NCBInr database (downloaded on May 20, 2008) using the Mascot software package (version 2.2) (Matrix Science).

References

1. Villanueva J, Philip J, Entenberg D et al. Serum peptide profiling by magnetic particle-assisted, automated sample processing and MALDI-TOF mass spectrometry. Anal Chem 2004; 76: 1560-1570.

2. Hayashida Y, Honda K, Osaka Y et al. Possible prediction of chemoradiosensitivity of esophageal cancer by serum protein profiling. Clin Cancer Res 2005; 11: 8042-8047.
